# *Causonissessilifolia* (Vitaceae), a new species from Thailand

**DOI:** 10.3897/phytokeys.185.75570

**Published:** 2021-11-15

**Authors:** Anna Trias-Blasi, Manop Poopath, Li-Min Lu, Gaurav Parmar

**Affiliations:** 1 Royal Botanic Gardens, Kew, Richmond, UK Royal Botanic Gardens, Kew Richmond United Kingdom; 2 Forest Herbarium (BKF), Department of National Parks, Wildlife and Plant Conservation, Bangkok, 10900, Thailand Department of National Parks, Wildlife and Plant Conservation Bangkok Thailand; 3 State Key Laboratory of Systematic and Evolutionary Botany, Institute of Botany, Chinese Academy of Sciences, Beijing, 100093, China Institute of Botany, Chinese Academy of Sciences Beijing China; 4 National Botanical Garden, Godawari, Lalitpur, Nepal National Botanical Garden Lalitpur Nepal

**Keywords:** *Causonis*, Thailand, new taxon, taxonomy

## Abstract

A new species, *Causonissessilifolia*, from Thailand is described, based on morphological and phylogenetical methods. A full description, conservation assessment, a key, images and phylogenetic tree are provided. Diagnostic characters for this species are sessile leaves that are sometimes opposite and inflorescence insertion interfoliar.

## Introduction

The genus *Cayratia* Juss. in the broad sense (s.l.) has consistently been found to be paraphyletic (Wen et al. 2007; Trias-Blasi et al. 2012; Lu et al. 2013; Parmar et al. 2021). To maintain the monophyly within Vitaceae, the species in Cayratiasect.Discypharia Suess. (Suessenguth, 1953; Latiff, 1981) which was later treated as Cayratiasubg.Discypharia (Suess.) C.L.Li (Li 1996, 1998; Chen et al. 2007), were placed in the newly-re-instated genus *Causonis* Raf. (Wen et al. 2013; Parmar et al. 2021). This genus can be separated from the *Cayratia* s.l. as it lacks a distinct membrane enclosing the ventral infolds in seeds (Wen et al. 2013; Parmar et al. 2021). *Causonis* can be distinguished from other Vitaceae genera by plants being hermaphroditic, flowers 4-merous, inflorescences mostly axillary, but sometimes pseudo-axillary.

*Causonis* comprises about 16 species and four varieties and is found in tropical, subtropical and temperate Asia to Australia including the Pacific Islands (Parmar et al. 2021). Trias-Blasi and Parnell (2020) reported two *Causonis* species in Thailand, but the specimen from Nakhon Sawan in the northern floristic region of Thailand, was found to be morphologically distinct from other species reported from the region.

**Figure 1.**
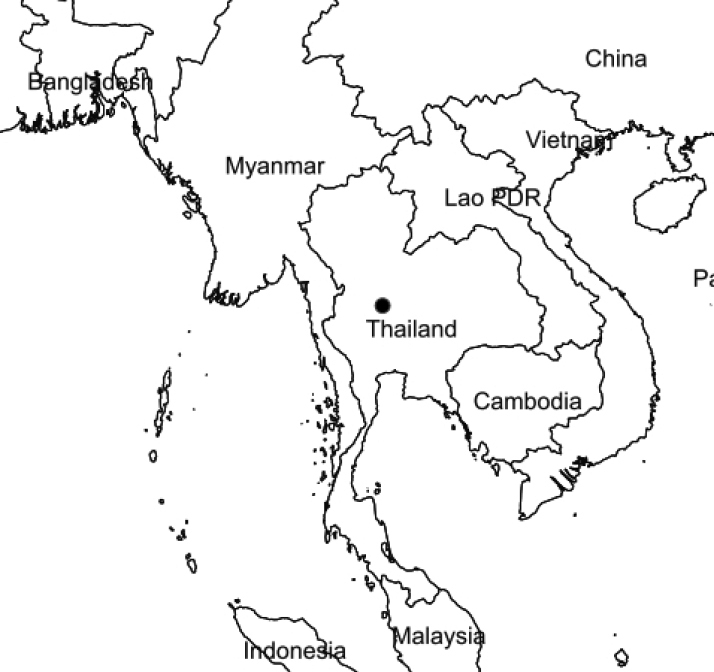
Map of the specimen collected (black circle).

In autumn 2019, an expedition was conducted in a 5-hectare area adjacent to Kriangkrai River in the Nakhon Sawan Province (Fig. 1), because of concerns relating to high levels of deforestation in the lowland floodplain forests in Thailand. Additionally, very few specimens had been collected from this habitat. Generally, these areas are flooded every year during September-November when the water increases by 1–3 metres. This species was found in an open area along the riverbank.

## Methods

This study is based on the material collected in October 2019 in Nakhon Sawan. Morphological characters were studied using a hand lens (30–60× magnification) and stereomicroscope and documented by photography. Collected specimens were thoroughly compared with protologues and types of all *Causonis* species occurring in Thailand and neighbouring regions. Additionally, herbarium material of *Causonis*, deposited in K, was studied (herbarium codes according to Thiers 2021). Herbarium vouchers for this study are deposited in BKF. The description follows the style and level of details outlined in Trias-Blasi and Parnell (2020), while the general terminology is based on Beentje (2016). The conservation assessment is based on the most recent version of the guidelines of IUCN Standards and Petitions Subcommittee (IUCN 2012).

Genomic DNA for the new taxon was extracted from silica-gel-dried leaf material using DNeasy Plant Mini Kit Qiagen (Qiagen, Hilden, Germany) following manufacturers’ protocols. All other sequences for different taxa were downloaded from GenBank. The DNAs were amplified for four chloroplast loci (*atpB-rbcL*, *trnC-petN*, *trnH-psbA* and *trnL-F*) following the protocols in Lu et al. (2018). The PCR products were purified and examined on a 1% agarose gel before being sent to Majorbio Company in Beijing, China, for sequencing on a Roche 454 sequencer with the same PCR amplification primers using a standard GS FLX Titanium sequencing kit XL+ (454 Life Sciences, Branford, CT, USA). The voucher specimens and the sequences’ GenBank accession numbers are provided in Table 1.

Geneious 8.1.9 was used to assemble forward and reverse sequences (Kearse et al. 2012). Geneious was also used to edit contiguous sequences and check chromatograms for base validation. Following that, sequences were aligned using MAFFT 1.3.1 (Katoh et al. 2002) and then manually adjusted in Geneious. On the CIPRES Science Gateway (Miller et al. 2010), phylogenetic analyses were first performed for individual DNA loci using the Maximum Likelihood (ML) approach in RAxML-HPC2 on XSEDE (8.2.12) using the GTR + G model with 1000 bootstrap replicates (Stamatakis 2014). Single tree analyses did not detect well-supported topological conflicts amongst individual DNA loci (i.e. ML BS < 70%; Hillis and Bull 1993). As a result, for further phylogenetic analyses, the four chloroplast DNA loci were concatenated. For the four chloroplast (4cp) dataset, partitioned ML and Bayesian Inference (BI) analyses were performed and the best fitting models for individual data partitions were selected using PhyML 3.0 (Guindon et al. 2010) with the Akaike Information Criterion (AIC). The nucleotide substitution model GTR + G was found to be the most suitable for *trnC-petN* and *trnL-F* and HKY85 + G for *atpB-rbcL* and *trnH-psbA*. MrBayes 3.2.6 was used to conduct Bayesian analysis on the CIPRES Science Gateway (Ronquist et al. 2012). For a total of 10,000,000 generations, four Markov Chain Monte Carlo analyses were conducted, with one tree sampled every 1,000 generations. The standard deviation between the split frequencies was found to be less than 0.01, indicating that enough generations had been completed. Following the burn-in of the first 25% of trees, the remaining trees were used to determine a 50% majority-rule consensus tree and posterior probabilities (PP). The trees obtained from ML and BI were analysed for topological conflicts through FigTree v.1.4.4 (Rambaut 2018).

## Taxonomic treatment

### Causonis sessilifolia

Taxon classificationPlantaeVitalesVitaceae

Trias-Blasi & G.Parmar sp. nov.

65DE1A62-4FC7-5AF2-8282-63ECDE9D077D

urn:lsid:ipni.org:names:77222603-1

#### Diagnosis.

Morphologically, *Causonissessilifolia* and *Causonisjaponica* (Thunb. ex Murray) Raf. share similarity in possessing 5-foliolate leaves, but the former taxon has sessile leaves and 2–5-furcate tendrils (vs. petiolate leaves and 2–3-furcate tendrils in *C.japonica*).

**Table 1.** Voucher information and GenBank accession numbers for the sequences used in this study.

Taxon	Voucher No.	Locality	*atpB-rbcL*	*trnC-petN*	*trnH-psbA*	*trnL-F*
*Causonisaustralasica* L.M.Lu & Jackes	*AU015* (PE)	Australia, Queensland	MW408585	MW408375	MW408696	MW408491
*Causonisclematidea* (F.Muell.) Jackes	*Wen 12184* (US)	Australia, New South Wales, Sydney (cult.)	KC166297	KC166475	KC166552	KC166625
*Causoniscorniculata* (Benth.) J.Wen & L.M.Lu	*YSL4758* (PE)	China, Taiwan	MW408551	MW408342	MW408665	MW408460
*Causonisdaliensis* (C.L.Li) G.Parmar & L.M.Lu	*VN2014116* (PE)	Vietnam, Lam Dong	MW408540	MW408333	MW408654	MW408450
*Causonisfugongensis* (C.L.Li) G.Parmar & L.M.Lu	*CPG36648* (PE)	Myanmar, Kachin	MW408564	MW408355	MW408676	MW408473
Causonisjaponica(Thunb.)Raf.var.japonica	*Wen 8537* (US)	Japan, Chiba	KC166313	KC166488	KC166564	KC166637
Causonisjaponicavar.pseudotrifolia (W.T.Wang) G.Parmar & J.Wen	*Wen 8085* (US)	China, Chongqing	AB234920	KC166498	KC166573	AB235006
*Causonismaritima* (Jackes) Jackes	*AU020* (PE)	Australia, Queensland	MW408567	MW408358	MW408679	MW408476
	*Wen 9403* (US)	China, Taiwan	KC166314	JF437193	JF437079	JF437299
*Causonismollis* (Wall. ex M.A.Lawson) G.Parmar & J.Wen	*CPG23617* (PE)	Vietnam, Vinh Phuc	MW408535	MW408328	MW408650	MW408445
*Causonissessilifolia* Trias-Blasi & G.Parmar	Poopath & Duangjai 2511	Thailand, Nakhon Sawan	OK338627	OK338628	OK338629	OK338630
Causonistimoriensisvar.mekongensis (C.Y.Wu ex W.T.Wang) G.Parmar & L.M.Lu	*CPG18926* (PE)	China, Yunnan	MW408580	MW408370	MW408692	MW408486
*Causonistrifolia* (L.) Mabb. & J.Wen	*CPG27533* (PE)	China, Yunnan	MW408575	MW408365	MW408687	–
	*CPG38701* (PE)	India, Kerala	MW408576	MW408366	MW408688	–
	*LA17* (PE)	Laos, Luang Namtha	MW408577	MW408367	MW408689	MW408484
	*Wen 7488* (US)	Thailand, Chiang Mai	KC166323	KC166500	KC166574	AB235007
	*CPG19178* (PE)	Indonesia, Bali	KC428757	KC428783	KC428800	KC428819
*Pseudocayratiapengiana* Hsu & J.Wen	*YSL4764* (PE)	China, Taiwan	MW408587	MW408377	MW408698	MW408493
*Pseudocayratiaspeciosa* J.Wen & L.M.Lu	*Wen 12026* (US)	China, Fujian	KC166377	–	KC166616	KC166682

“–” represents missing sequences.

#### Type material.

**Thailand. Northern floristic region**: Nakhon Sawan, Muang, Kriangkrai subdistrict, abandoned area at the bridge of Kriangkrai Canal, 15°44'40"N, 100°11'9"E, 23 October 2019, M. Poopath & S. Duangjai 2511 (holotype, BKF! (SN229663 (Fig. 5)), isotype BKF! (SN229662)).

#### Description.

**Figure 2.**
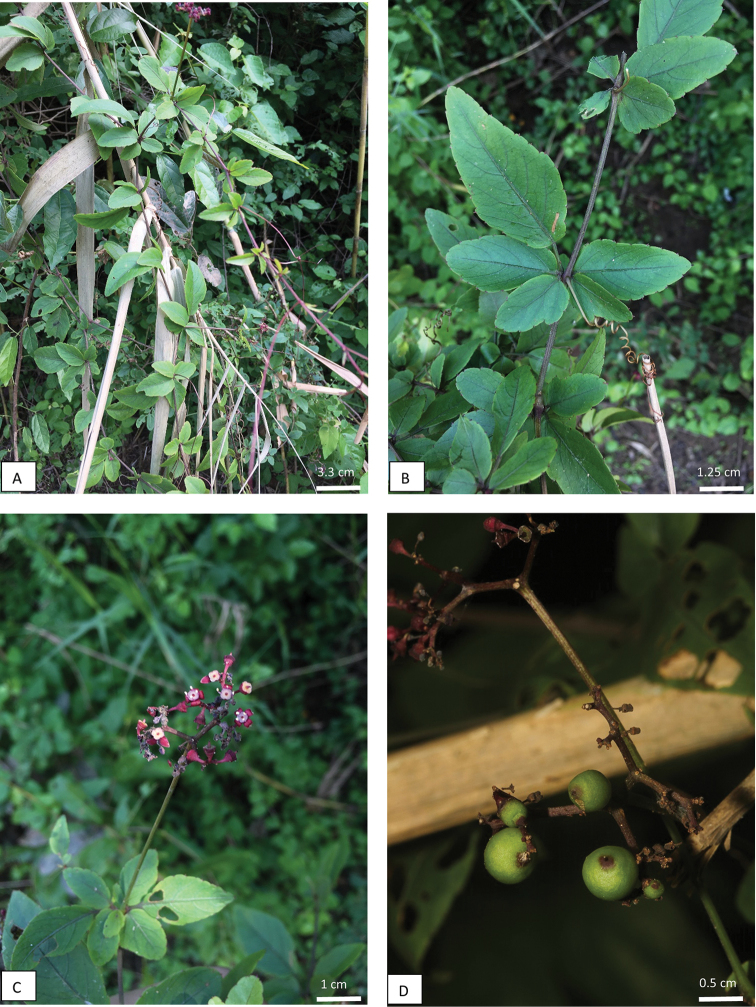
*Causonissessilifolia***A** habit **B** pedately 5-foliolate leaves **C** flowers and opposite leaves **D** fruits. Images: Sutee Duangjai and Manop Poopath.

Slender herbaceous climber. *Stem* cylindrical, 2–5 mm diameter, branched, glabrous, young stems purplish-green, hairy with some bent hairs to glabrous; tendril 2–5-furcate, slender, wiry, leaf-opposed, cylindrical, with a straight section, then bifurcating and coiling, 5–10 cm long, glabrous. *Leaves* compound, pedately 5-foliolate, alternate or opposite; petiole absent, central petiolule 0.5–1.5(–3) cm long, middle petiolules sessile, lateral petiolules 0.5–2 mm long, mostly glabrous, sometimes with bent hairs; central leaflet blade lanceolate to slightly rhombic, 2–5 by 1–2.5 cm, base cuneate; middle leaflet blade lanceolate, 1–3 by 0.5–1.5 cm; lateral leaflet blade lanceolate often with a single asymmetric acute lobe, 0.5–1.75 by 0.2–1.25 cm, base cuneate; margin broadly denticulate, apex acuminate to mucronate; adaxial and abaxial sides glabrous, mid-rib raised on upper surface, each leaflet with 2–5 pairs of lateral veins, if leaflet lobed then lower lateral vein may be more distinct. *Inflorescence* ramified, interfoliar or pseudo-terminal, mostly dividing dichotomously, with numerous ramifications, 0.7–2 by 1–2.5 cm, lax, erect; peduncle 2–8 cm long, glabrous, pedicel 0.75–2 mm long, glabrous. Buds ovoid, 1–2 by 1–2 mm. *Calyx* cupuliform, entire, margin sinuate, 0.5–1 by 1–1.25 mm, glabrous, dark red. *Corolla* petals 4, ovate, 1.5–2 by 1.25–2 mm, apex cucullate, glabrous, dark red. *Stamens* 4; filaments flattened, broadening at the base, 1 mm long, cream; anthers elliptic, medifixed, 0.5–1 mm long, cream. *Ovary* adnate to the disc; disc with 4 distinct lobes, cupular, 0.5 by 1.5 mm, glabrous, dark red outside and whitish inside. *Style* conical, 0.5 mm long; stigma inconspicuous, dark red. *Fruit* berry, globose, 4–7 mm in diameter, glabrous, smooth, green. *Seeds* 2, 4–5 by 3 mm, adaxial side with two faces, abaxial side convex and ovate with a linear chalazal knot (Fig. 2).

#### Phenology.

Flowering and fruiting in October.

**Figure 3.**
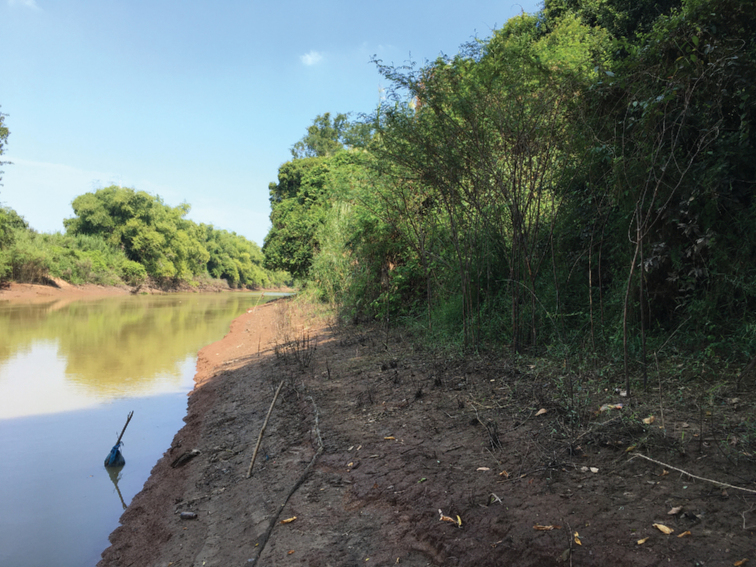
*Causonissessilifolia*. Habitat. Images: Manop Poopath.

#### Etymology.

The specific epithet “*sessilifolia*” refers to the sessile leaves of the taxon.

**Figure 4.**
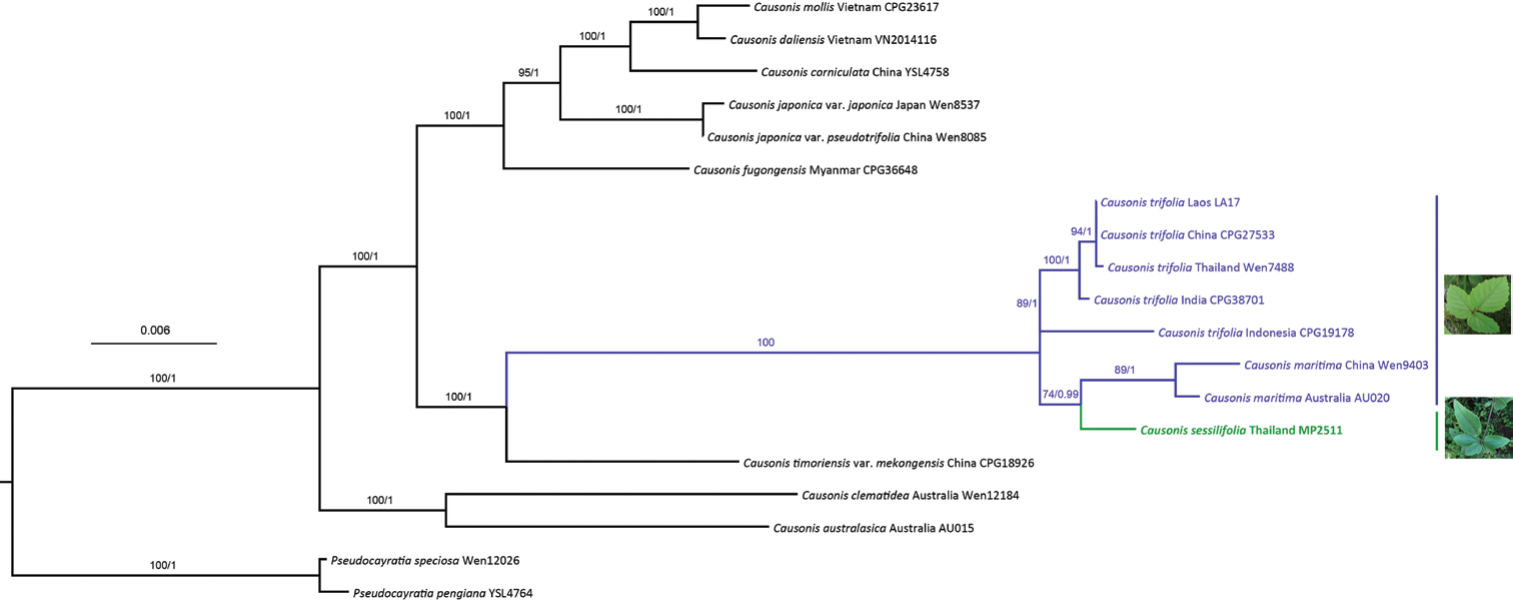
Phylogeny of selected species of *Causonis* including *C.sessilifolia*, based on the combined chloroplast dataset (*atpB-rbcL, trnC-petN, trnH-psbA* and *trnL-F*). Maximum Likelihood bootstrap values and Bayesian posterior probability values are indicated above branches respectively.

#### Distribution and habitat.

Thailand (Northern floristic region, Nakhon Sawan). Lowland floodplain forest, along the bank of canal in open areas; 30 m alt. (Figs 1, 3).

**Figure 5.**
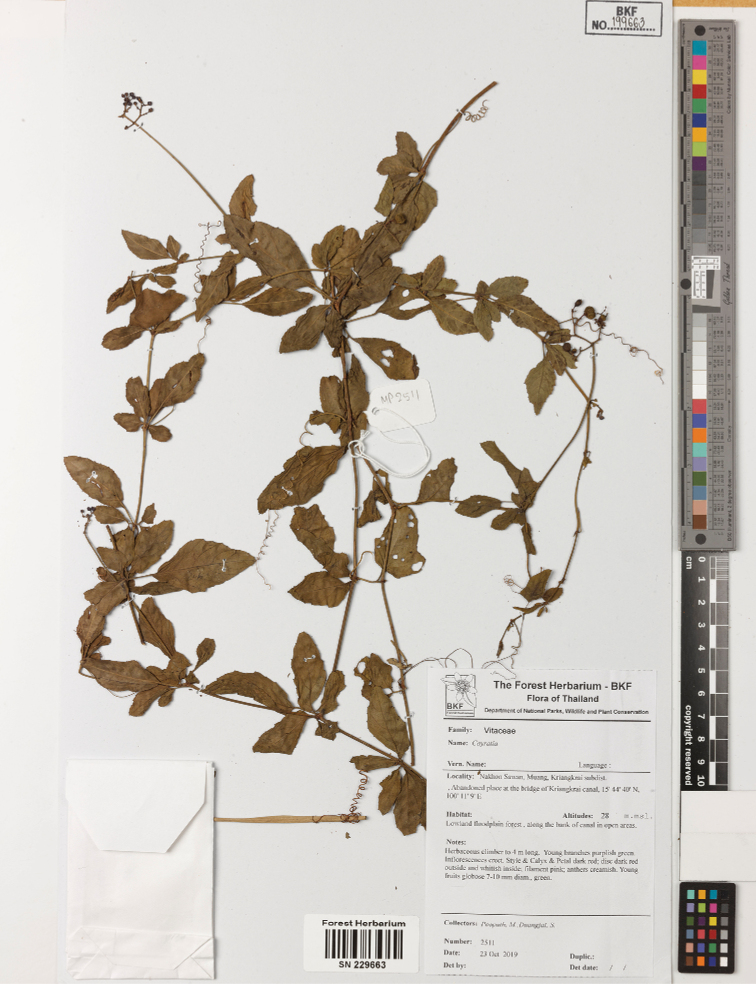
Holotype of *Causonissessilifolia*.

#### Conservation status.

This species is only known from the type locality and, therefore, has an Area of Occupancy (AOO) and Extent of Occupancy (EOO) of 4 km^2^. This suggests that it might be Critically Endangered as the AOO is less than 10 km^2^ and is only found in one location. The species has been found to grow outside any protected areas and in an abandoned area next to a canal. This could mean the species is more vulnerable than others as it is unprotected. Additionally, all the surrounding areas are used for agriculture and, therefore, it is likely this forested area might also be transformed for this use. Due to this threat, the restricted AOO and number of locations, we think that this taxon could be driven to being Critically Endangered or Extinct in a very short time; therefore, we assess the taxon as VUD2 (IUCN 2012).

#### Taxonomic remarks.

Phylogenetically, this pedately 5-foliolate leaved species lies in a clade previously known with species of exclusively trifoliolate leaves such as *Causonistrifolia* (L.) Mabb. & J.Wen and *Causonismaritima* (Jackes) Jackes (Parmar et al. 2021), but *C.sessilifolia* lacks the petiolate leaves found in *C.trifolia* and *C.maritima*. In particular, this species is phylogenetically most closely related to *C.maritima* (ML BS = 74%; Bayesian PP = 0.99; Fig. 4), but morphologically differs from it in having pedately 5-foliolate leaves (vs. trifoliolate), leaves sometimes opposite (vs. leaves always alternate), leaves sessile (vs. leaves petiolate), inflorescence insertion interfoliar (vs. axillary), calyx and corolla glabrous (vs. hairy).

### Key to *Causonis* in Thailand (including *Causonismaritima*)

**Table:** 

1	Leaves 3-foliolate	**2**
–	Leaves 5-foliolate	**3**
2	Tendrils 2–3-furcate, tips without adhesive disc; hooked hairs confined to mid-vein on adaxial surface	**1. *C.maritima***
–	Tendrils 3–5-furcate, tips with adhesive disc; hairs all over abaxial and adaxial surfaces	**2. *C.trifolia***
3	Petiole present; inflorescence leaf-opposed, pseudoaxillary or axillary	**3. *C.japonica***
–	Petiole absent; inflorescence interfoliar	**4. *C.sessilifolia***

## Supplementary Material

XML Treatment for Causonis sessilifolia
